# A psychiatrist's perspective from a COVID-19 epicentre: a personal account

**DOI:** 10.1192/bjo.2020.83

**Published:** 2020-09-09

**Authors:** Isabella Pacchiarotti, Gerard Anmella, Giovanna Fico, Norma Verdolini, Eduard Vieta

**Affiliations:** Bipolar and Depressive Disorders Unit, Institute of Neuroscience, Hospital Clinic, University of Barcelona, IDIBAPS, CIBERSAM, Spain; Bipolar and Depressive Disorders Unit, Institute of Neuroscience, Hospital Clinic, University of Barcelona, IDIBAPS, CIBERSAM, Spain; Bipolar and Depressive Disorders Unit, Institute of Neuroscience, Hospital Clinic, University of Barcelona, IDIBAPS, CIBERSAM, Spain; Bipolar and Depressive Disorders Unit, Institute of Neuroscience, Hospital Clinic, University of Barcelona, IDIBAPS, CIBERSAM, Spain; Bipolar and Depressive Disorders Unit, Institute of Neuroscience, Hospital Clinic, University of Barcelona, IDIBAPS, CIBERSAM, Spain

**Keywords:** COVID-19, SARS-CoV-2, psychiatry, mental health, telemedicine

## Abstract

**Background:**

The COVID-19 pandemic has and will have a huge impact on mental health, especially in countries that have been significantly affected, such as Spain.

**Aims:**

Here we aim to provide the perspectives of a group of psychiatrists from Barcelona, one of the epicentres of the pandemic so far, to highlight the potential fatality of a virus that caught us unaware and unprepared, and hopefully this article will be of aid to countries about to face the pandemic.

**Results:**

The unprecedented situations that we have been faced with so far have included reconfiguring hospitals and the redeployment of healthcare professionals, with flexibility and adaptability key to managing the overload in demand. This has led to healthcare professionals being exposed to extremely stressful situations and they have had impossible decisions to make that may have mental health consequences, some of which may be severe and long lasting.

**Conclusions:**

A rebound effect on mental health problems is to be expected in the medium and long term, especially for healthcare professionals and psychiatric patients, necessitating a strengthening of preventive approaches and policies for mental health along with a prompt reopening of mental health services. Ways to provide psychiatric healthcare in the immediate future need to be re-evaluated, and the development of telepsychiatry services is probably to be expected.

The Coronavirus disease 2019 (COVID-19) pandemic, with more than 12 million cases and 500 000 deaths reported worldwide,^1^ has and will have a huge impact on mental health, especially in those countries most affected, such as Spain.

Here we provide the perspectives of a group of psychiatrists from Barcelona, one of the epicentres of the pandemic so far, to highlight the potential fatality of a virus that has caught us unaware and unprepared, and we hope this article will be of value to countries about to face the pandemic.

## Results

### The COVID-19 outbreak: Europe unaware?

In December 2019, the novel severe acute respiratory syndrome coronavirus 2 (SARS-CoV-2) emerged in Wuhan, China.^2^ At the time of writing, almost 150 days later, more than 4 500 000 infections have been confirmed worldwide in the COVID-19 pandemic, with an approximate mortality of 7%.^3^ Worldwide a number of different public health responses have been used to attempt to contain the pandemic,^4^ with China imposing the first complete lockdown on the 23 January 2020, with 571 officially confirmed cases and 17 deaths.^5^

Despite the successful policies used in Asian countries to contain the spread of COVID-19, Europe failed to recognise the magnitude of the pandemic. Many European countries did not promptly deliver a coordinated response as the crisis was unfolding. Indeed, the government of Italy imposed lockdown on the 8 March 2020, with 5800 confirmed cases and 233 deaths^6^ and the Spanish government declared a state of emergency and imposed a lockdown on the 14 March 2020 with 6251 confirmed cases and 193 deaths.^7^ Many Spanish scientists have criticised the insufficient measures taken to contain the spread of the virus, claiming to cause Spain's move from partial to complete lockdown.^8^ This was probably because of a generalised underestimation of SARS-CoV-2 and overconfidence from European governments, which were sceptical about the initial need for state-of-emergency declarations. To add fuel to the fire, many social mass gatherings were held all over Europe in the weeks and days before Italy and Spain imposed lockdowns^9^ despite it being clear that the dynamics behind the virus's rapid exponential expansion worldwide had relied heavily on social gatherings. These events appear to have been conducted with astounding connivance and silence from the political sector. The later exponential escalation in cases and deaths in Italy and Spain leave to doubt if all this could have been prevented.

### Healthcare system upside-down: unprecedented measures for unprecedented situations

**Fig. 1:**
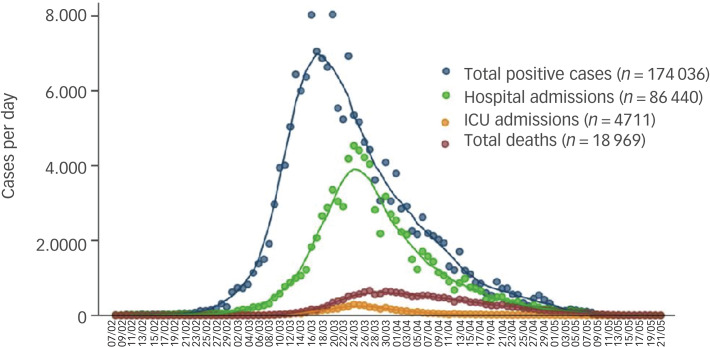
The epidemic curve for COVID-19 confirmed cases in Spain according to clinical severity.

**Fig. 2:**
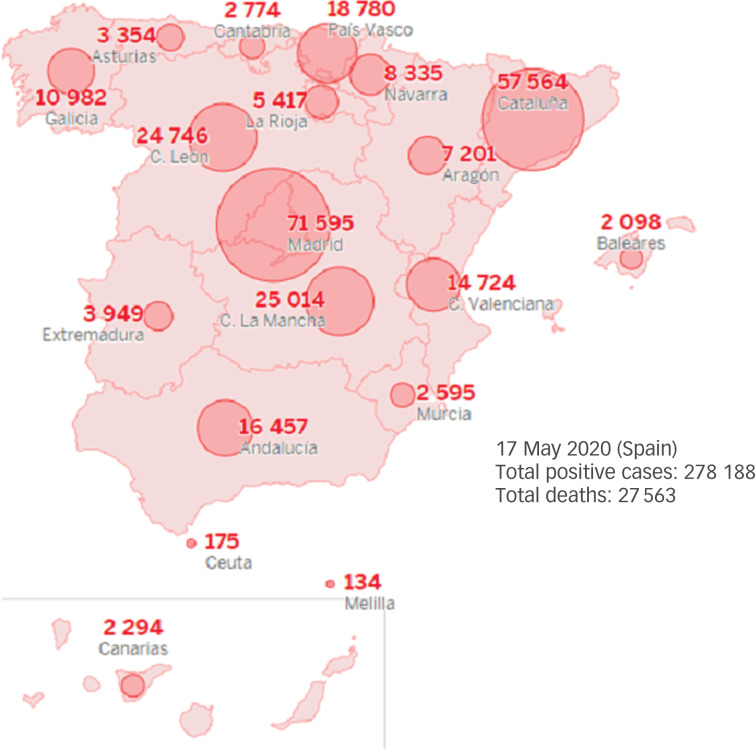
Geographical distribution by autonomous community in Spain for the total number of confirmed cases of COVID-19.

Source: data for COVID-19 cases reported by the Spanish Ministry of Health, Consumer Affairs and Social Welfare.^12,13^ Data updated on the 17 May 2020.

The aftermath of the probably insufficient preventive public health policies implemented by Italy and Spain has led to more than 240 000 cases and 28 000 deaths from COVID-19 in each of these countries, respectively, leading the world ranking (at the time of writing the first draft of this manuscript, 2 May 2020).^10^ The epidemic curve for confirmed cases of COVID-19 in Spain, according to clinical severity, can be seen in Fig. 1 and the geographical distribution of COVID-19 confirmed cases by autonomous community is reported in Fig. 2.

*n*, the cases per day are represented according to the date at the onset of symptoms, date of hospital admission and date of ICU admission, and deaths according to the official dates of death. Each line reports trends with moving averages for 7 days. Source: Informe n° 32. Situación de COVID-19 en España a 21 de mayo de 2020. Equipo COVID-19. Red Nacional de Vigilancia Epidemiológica (RENAVE). Centro Nacional de Epidemiología (CNE). Centro Nacional de Microbiología (CNM). Instituto de Salud Carlos III (ISCIII).^11^ Data updated on the 21 May 2020.

Several major public health measures were urgently adopted by most countries to reduce spread, including forced quarantine and restructuring of health systems, with the redeployment of healthcare professionals from all disciplines to COVID-19 units, following assistance requirements.^4^

This exponential growth in a matter of days put public healthcare systems under severe stress, with hundreds of new cases of complicated COVID-19 arriving in the emergency rooms of most hospitals with insufficient admission capacity and intensive care unit (ICU) beds in public health systems that were already usually working at nearly full capacity.

As a representative model, the Hospital Clínic of Barcelona has been ‘literally’ turned upside-down with the unceasing overflow of patients with COVID-19 arriving in the emergency room and requiring admission in the weeks after lockdown. The lack of enough room to admit these patients brought about the creation of a ‘crisis cabinet’ to lead a full restructuring, by transforming day hospitals, dialysis rooms and operating rooms into ICUs. A total of 42 ICU beds were occupied in only 48 h, and at the peak of the pandemic outbreak a total of 11 ICUs were active, with 110 occupied ICU beds. Moreover, the internal medicine and infectious diseases hospitalisation units were overcrowded and a total of 16 hospitalisation units were cleared by discharging less-severe hospitalised patients and converting these units into COVID-19 units.

Non-urgent surgical activities and out-patient medical appointments were postponed. Hospital hallways and unprepared hospitalisation rooms were provided with oxygen supplies and improvised into COVID-19 facilities, and libraries were transformed into medical equipment warehouses. Other unprecedented measures were also taken, such as refurbishing a nearby hotel to transform it into an extension of the hospital with the capacity to treat 300 patients with COVID-19 including fitting it out with oxygen supplies, a hospital pharmacy and an x-ray machine. In sum, the Hospital Clínic of Barcelona was completely reconfigured in a few days, tripling its ICU beds and attending, simultaneously, to a total of 900 in-patients with COVID-19: 600 in-patients (110 in ICU) and up to 300 on home hospitalisation (which includes home visits by a medical team and tele-monitoring).^14^

### Healthcare professionals: the high cost of good clinical practice

Besides the aforementioned structural changes to healthcare facilities, healthcare professionals from all disciplines have been involved in the restructuring in several ways: many have been redeployed in novel COVID-19 hospitalisation units or even transferred to different cities based on assistance requirements, and many healthcare specialists have had to rebrand and update themselves into the infectious-respiratory field of medicine because of the lack of sufficient trained professionals.^4^

Thus, healthcare professionals, especially those on the frontline, who have been at greater risk of being infected,^15^ have been exposed to unprecedented stress, facing increased workload and great emotional burden. Their concerns about the risks of infection and developing complications, and the constant fear of spreading the virus to their families at home and colleagues, has exposed them to greater isolation measures with worse psychological outcomes.^16,17^ Indeed, healthcare professionals are highly vulnerable to experiencing physical exhaustion, fear, emotional disturbance, stigmatisation, insomnia, depression and anxiety, distress, substance use, post-traumatic stress disorder (PTSD) symptoms, and even suicide.^18–21^ A recent study on frontline healthcare professionals in Italy, reported rates of almost 50% for PTSD symptoms, 25% for severe depression and 20% for anxiety and insomnia.^22^ Additionally, a potential increase in brief reactive psychosis in healthcare professionals because of the COVID-19 outbreak has recently been reported.^23^ This emphasises the need for urgent mental health preserving strategies for healthcare professionals during the current pandemic.^24^

Healthcare professionals have been also implicated as ‘super-spreaders’ with nosocomial origins of infection being a key component in community propagation of SARS-CoV-2. The lack of personal protective equipment (PPE), such as gloves or masks, has been a recurring problem in many regions of Italy and Spain during the first phase of the crisis and raised the ethical dilemma of beneficence and non-maleficence. When not enough PPE is available, should healthcare professionals continue to offer treatment on deontological grounds or refuse on the grounds that becoming a ‘super-spreader’ would be more harmful to society? This is still a matter of debate, and one in which healthcare professionals should preserve their right to make their own personal decision.^25^ However, in real-world practice during this pandemic, and especially in countries like Spain or Italy, it would be inconceivable not to provide treatment for a net societal good, so this important debate has taken place on a theoretical basis and has been so far been drowned out by healthcare authorities claiming the principle of beneficence, and most professionals have felt compelled to work sometimes under unprotected conditions.

Several members of our team, including some of the authors, were relocated and redeployed to aid with direct or indirect medical attention for patients with COVID-19 and one of them was exposed in the frontline (in a newly converted COVID-19 hospitalisation unit). The uncertainty surrounding every aspect of the COVID-19 outbreak was a key aspect contributing to suffering. We did not know the magnitude of the catastrophe that was to be expected, nor the length of time that we were going to be in that situation, or even sometimes, what to do, what to tell our families, or how to support our colleagues and loved ones. At some points, while blinded by uncertainty, only the quote by Winston Churchill shed some light: ‘Now this is not the end. It is not even the beginning of the end. But it is, perhaps, the end of the beginning’.^26^

Several members of our team experienced the burden of having to take on major responsibilities because of unprecedented, and sometimes impossible, decisions such as: how to organise the acute psychiatric hospital unit with insufficient COVID-19 detection and protection methods both for in-patients and healthcare professionals. This huge emotional load, which lasted for more than 2 months during the peak of the pandemic, has taken a toll on many members of our team, who have experienced stress-related symptoms. However, subsequent evaluations from many team members agree that the supportive and collaborative atmosphere that was created back then has been key to preventing many healthcare workers from collapse.

### Mental health consequences in the general population: dear lockdown

The current pandemic is also leading to negative psychological effects in the general population.^27,28^ Among stressors, fear of infection, a longer quarantine, isolation or movement restrictions, lack of information, financial loss, inadequate supplies and stigma all contribute to deterioration in psychological outcomes. At such times, keeping the general public informed about protocols is essential and supplies should be ensured.^29^ Lockdown, isolation, movement restriction and/or quarantine, may have a substantial and long-lasting negative psychological impact on the general population, so it needs to be handled carefully. The reported negative psychological effects include feelings of anger, frustration, boredom and guilt, post-traumatic stress symptoms, confusion, insomnia and suicide. However, many people may present with persistent and/or delayed effects in the form of anxiety disorders and depression.^30^

More severe mental health problems may arise from the current crisis as stressful events in adult life have been shown to increase up to threefold the risk of psychosis in previously healthy individuals^31^ and preliminary evidence points to an increase in first-episode psychosis in the months since the outbreak of the pandemic.^32^ Recent evidence from a hospital in southern Spain suggests, over a short period of time, there has been an increase in brief reactive psychotic disorders in previously healthy populations directly triggered by stress related to the pandemic.^33^

In addition to the effects caused by lockdown, many people are likely to experience the effects of another particular feature of the current pandemic – the cruelty of isolation from caring for relatives who are dying because of their infection with COVID-19. This carries intrinsic suffering both for dying people and their close relatives and may leave sequelae in the form of pathological mourning. To prevent it, communication between patients and their families should be offered as frequently as possible. In this regard, psychologists are playing a fundamental role in many hospitals. In our unit, the psychology team has led an initiative to facilitate communication between patients with COVID-19 and their families by video calls with the nursing teams. Another initiative has been the psychological team working in shifts every day providing supportive care for patients with COVID-19 and their relatives. They have also issued several recommendations for dealing with grieving families and are working together with several healthcare teams for training, but also to provide support for healthcare professionals struggling with the whole situation, in collaboration with the consultation-liaison psychiatry (CLP) unit. Preventive strategies must be prepared to help identify high-risk individuals in the early stages of complicated grief that emerge from the current pandemic.^34^

### Psychiatric populations: where and how are our patients?

People with mental disorders are both direct and indirect victims of the pandemic.^35,36^ They may be more susceptible to infections, including pneumonia^37^ for a variety of reasons including cognitive impairment, negligence relating to risks, confinement conditions on psychiatric wards, restrictions in regular out-patient evaluations, pharmacological treatment and unequal access to healthcare settings.

At the peak of the pandemic in Spain, as mentioned above, many specialties were forced to reconfigure their wards in order to treat patients with COVID-19. Hospital psychiatric units were not unaffected, and several acute in-patient units have been converted and others have seen their admission capacity reduced. In our centre, the child and adolescent in-patient psychiatric unit with 21 beds was converted into a COVID-19 unit; the adult psychiatric in-patient unit's capacity for admission was reduced by 50% (to isolate and test susceptible patients for SARS-CoV-2 infection); the psychiatric emergency room was converted for terminally ill patients with COVID-19 who were not candidates for ICU; and the day hospital with a capacity for 16 patients was closed. Although we have been trained to operate on the bases of the principles of distributive justice, equity, autonomy and recovery, we were forced to give assistance prioritising those with the most severe conditions.

With these shortages in several (often considered essential) units providing psychiatric medical attention, one would have expected an increase in the psychiatric demand in the emergency room. However, there was a clear reduction in the number of emergency room visits, as well as hospital admissions for patients with psychiatric disorders. This has posed the question: ‘where and how are our patients?’.

This decrease in attendances has also occurred for other non-psychiatric medical conditions, such as strokes and heart attacks, where individuals are seen in the emergency room much later than usual and with more serious symptoms. The plausible explanations for the drop in demand for medical attention include the fear of infection and the mandatory confinement. However, even with the drop in demand, we find ourselves in a highly stressful situation with lockdown being a source of stress and anxiety, and patients with psychiatric pathologies are particularly vulnerable and may present relapses. This situation can be particularly stressful for children with neurodevelopmental disorders, such as autism spectrum disorders or intellectual disability that need a daily routine and may be particularly affected if they or their caregivers become infected because of the need for isolation.^38^ Patients with schizophrenia, bipolar disorder, and other severe mental disorders may also be more vulnerable and individuals with substance use disorders may experience withdrawal syndrome because of difficulty obtaining drugs, whereas other addictions, such as alcoholism, smoking and online gambling may worsen.^39^

Furthermore, patients with mental disorders are often stigmatised^40^ and it has also been reported^41^ that COVID-19 can be associated with a higher risk of stigmatisation.^42^ Thus, psychiatric patients with COVID-19 could be subjected to a double stigma, that might lead to reduced access to medical services, social isolation and worst health outcomes.^43^

### Towards a new psychiatry in times of pandemic

As psychiatrists, we are exposed to new challenges. First, many psychiatrists have been recruited as general hospital doctors in healthcare teams dealing with COVID-19. This has reminded us of the value of basic medical training for those working in psychiatry. Our general medical knowledge is essential and may be required, thus psychiatry should be recognised as an equally considered medical specialty.^30^

In psychiatric practice, to compensate for the shortages in several psychiatric units, other alternatives have been strengthened: mental health home care and home hospitalisation care (which includes home visits by a medical team and tele-monitoring) has been proposed as an alternative in order to avoid psychiatric hospital admissions, to ensure regular administration of long-acting injectable antipsychotic medications and to provide continuous monitoring of laboratory parameters during psychopharmacological treatment, including the tests required for patients treated with lithium and clozapine.^44^

Another compensatory measure that has been adopted in psychiatry as well as in many medical specialties is a transition to telemedicine with out-patient tele-visits (via telephone, chat or video call).^45^ Mental health seems a good candidate for telemedicine as a physical examination is not always required.^46^ So far, during the pandemic, it has been a practical alternative and many problems have been solved this way, especially for patients who have been followed-up for some time already and who are able to maintain a close relationship with their therapists this way.^47^ However, much psychopathological information might be lost and new consultations, in particular, are hampered by telepsychiatry. In the post-COVID-19 scenario, mental health is probably going to benefit from the increasingly important role of m-health (mobile health),^48,49^ which may serve as an instrument for case management and patient empowerment.^50^

Following the hospital conversions, CLP units have gained prominence. Our CLP unit (usually consisting of two psychiatrists) has been reinforced with more than seven psychiatrists at the peak of the pandemic. The unit faced challenges in the psychopharmacological management of patients relating to potential interactions of psychotropic medications with experimental COVID-19 therapies.^51^ As a result of the complexity of real-world clinical scenarios and the lack of case-centred recommendations, our CLP unit has issued practical recommendations for the psychopharmacological management of the most representative case scenarios that we have identified regarding patients who were admitted to hospital with COVID-19 who had psychiatric disorders, based on existing literature and clinical experience.^52^

Another matter of concern has been what to do with patients requiring a psychiatric admission but who are also infected with SARS-CoV-2 (but who do not require admission because of respiratory problems). This has led to strict control of patients who need admission for psychiatric reasons using polymerase chain reaction-based tests and admissions into either (a) ‘clean’ psychiatric in-patient units; (b) ‘COVID-19’ psychiatric units, or (c) non-psychiatric COVID-19 hospital units with the support of CLP units. When a patient requires a psychiatric admission and is also infected by SARS-CoV-2, this poses several problems relating to the need for isolation, which may be difficult for patients with those psychiatric conditions that include behavioural changes (such as disorganised behaviour and altered judgement during severe psychotic episodes), or those who disrespect social distancing in manic states etc. These situations may pose additional risk for patients and healthcare professionals.

## Discussion

### Future perspectives: making mental healthcare essential

**Fig. 3:**
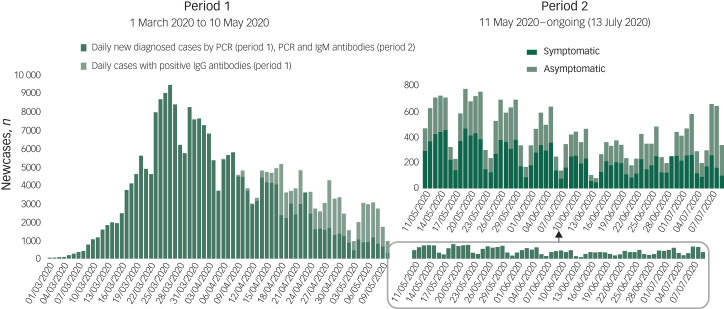
Daily confirmed COVID-19 cases in Spain (updated on 09 July 2020).

**Fig. 4:**
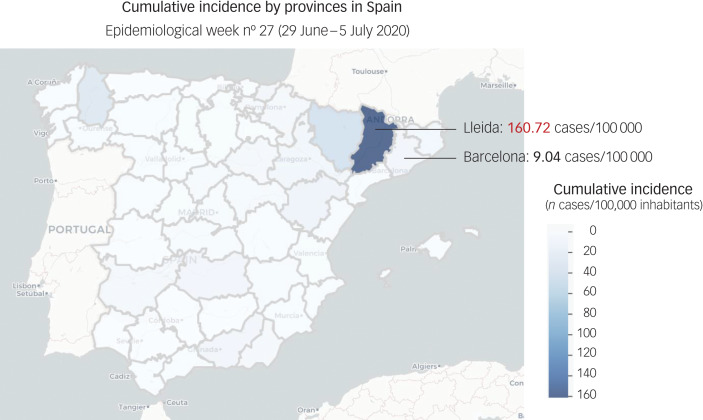
Cumulative incidences by province in Spain for epidemiological week 27 (29 June to 5 July 2020).

Predictive models suggest that after this current, most severe pandemic, wave recurrent outbreaks of SARS-CoV-2 will probably occur. In Spain, after the peak of the pandemic (Fig. 3), several COVID-19 resurgences have been arising on a local basis (Fig. 4). Therefore, unless critical care capacity is increased substantially or a vaccine becomes available, prolonged or intermittent social distancing has been predicted to be necessary into 2022. Moreover, the duration of acquired immunity to SARS-CoV-2 is unclear and longitudinal serological studies are needed. Predictions suggest that a resurgence in contagion could be possible as late as 2024.^55^

The new daily confirmed cases have been divided into two periods according to approval on 11 May 2020 of the new diagnostic, surveillance and control strategy for the ‘transition’ phase of the COVID-19 pandemic. On the left, the first period (from 1 March 2020 to 10 May 2020) showing daily newly diagnosed cases (by polymerase chain reaction (PCR)) and daily cases with positive immunoglobulin (Ig)G antibodies (presumed immunity). On the right, the second period (from 11 May 2020 ongoing until the time of writing on 13 July 2020) showing daily newly diagnosed cases (by PCR or positive IgM antibodies) divided by symptom presentation. Source: Actualización n^o^ 160. Enfermedad por el coronavirus (COVID-19). Ministerio de Sanidad - Gobierno de España.^52^ Data updated on the 9 July 2020.

After the peak of the pandemic in Spain, lockdown and restriction measures have been progressively withdrawn. The state of emergency expired on 21 June 2020, and each autonomous community has been responsible for the detection and management of COVID-19 resurgences since then, which have been arising continuously. The figure shows the focuses of COVID-19 resurgence during epidemiological week 27. Catalonia, in the north-eastern part of the map, includes four provinces (Barcelona, Lleida, Girona and Tarragona). One of them (Lleida) experienced a COVID-19 resurgence in week 27 (160.72 cases/100 000 compared with 9.04 cases/100 000 in Barcelona in the same week), and restriction measures have been applied accordingly by the Catalan Government. Source: Department of Health. Spanish Government. Red Nacional de Vigilancia Epidemiológica (RENAVE). Centro Nacional de Epidemiología (CNE). Centro Nacional de Microbiología (CNM).^54^ Data updated on the 10 July 2020.

There has been a coordinated response in mental healthcare so far and many of the problems faced have been solved along the way. However, despite all efforts, even if most of the mental health issues caused by the current pandemic may be mild and transient, because of the huge impact in different magnitudes put on the whole population, many will have long-lasting mental health consequences. A rebound effect on mental health problems is expected in the medium and long term, both in people without pre-existing mental health problems (especially in populations at risk, such as healthcare professionals) and in psychiatric patients. This foreseeable increase in demand for psychiatric care highlights the necessity of strengthening preventive approaches and policies in mental health services, including early identification, early treatment and close long-term monitoring of people with the most severe mental health problems; services that have often been marginalised.^34^

The ongoing process of change in the public health system and the way we interact is raising new questions relating to mental health. As soon as possible after the peak of the pandemic it will be imperative to progressively reopen all mental health resources that have been closed or reduced, to reinforce in-patient and out-patient mental health and primary care services,^30^ and to model novel interventions to tackle the current crisis and its consequences.^50^

## Data Availability

Data availability is not applicable to this article as no new data were created or analysed in this study.

## Supplementary material

For supplementary material accompanying this paper visit http://dx.doi.org/10.1192/bjo.2020.83. click here to view supplementary material
